# Runs of homozygosity in the Italian goat breeds: impact of management practices in low-input systems

**DOI:** 10.1186/s12711-021-00685-4

**Published:** 2021-12-11

**Authors:** Matteo Cortellari, Arianna Bionda, Alessio Negro, Stefano Frattini, Salvatore Mastrangelo, Elisa Somenzi, Emiliano Lasagna, Francesca M. Sarti, Elena Ciani, Roberta Ciampolini, Donata Marletta, Luigi Liotta, Paolo Ajmone Marsan, Fabio Pilla, Licia Colli, Andrea Talenti, Paola Crepaldi

**Affiliations:** 1 Dipartimento di Scienze Agrarie e Ambientali - Produzione, Territorio, Agroenergia, Università degli Studi di Milano, Milan, Italy; 2 Dipartimento di Scienze Agrarie, Alimentari e Forestali, Università degli Studi di Palermo, Palermo, Italy; 3 Dipartimento di Scienze Animali, Della Nutrizione e Degli Alimenti and BioDNA Centro di Ricerca Sulla Biodiversità e Sul DNA Antico, Università Cattolica del Sacro Cuore, Piacenza, Italy; 4 Dipartimento di Scienze Agrarie, Alimentari e Ambientali, Università degli Studi di Perugia, Perugia, Italy; 5 Dipartimento di Bioscienze Biotecnologie e Biofarmaceutica, Università degli Studi di Bari, Bari, Italy; 6 Dipartimento di Scienze Veterinarie, Università di Pisa, Pisa, Italy; 7 Dipartimento di Agricoltura, Alimentazione e Ambiente, Università degli Studi di Catania, Catania, Italy; 8 Dipartimento di Scienze Veterinarie, Università degli Studi di Messina, Messina, Italy; 9 Dipartimento Agricoltura, Ambiente e Alimenti, Università degli Studi del Molise, Campobasso, Italy; 10 The Roslin Institute, University of Edinburgh, Edinburgh, UK

## Abstract

**Background:**

Climate and farming systems, several of which are considered as low-input agricultural systems, vary between goat populations from Northern and Southern Italy and have led to different management practices. These processes have impacted genome shaping in terms of inbreeding and regions under selection and resulted in differences between the northern and southern populations. Both inbreeding and signatures of selection can be pinpointed by the analysis of runs of homozygosity (ROH), which provides useful information to assist the management of this species in different rural areas.

**Results:**

We analyzed the ROH distribution and inbreeding (*F*_ROH_) in 902 goats from the Italian Goat Consortium2 dataset. We evaluated the differences in individual ROH number and length between goat breeds from Northern (NRD) and Central-southern (CSD) Italy. Then, we identified the signatures of selection that differentiate these two groups using three methods: ROH, ΔROH, and averaged *F*_ST_. ROH analyses showed that some Italian goat breeds have a lower inbreeding coefficient, which is attributable to their management and history. ROH are longer in breeds that are undergoing non-optimal management or with small population size. In several small breeds, the ROH length classes are balanced, reflecting more accurate mating planning. The differences in climate and management between the NRD and CSD groups have resulted in different ROH lengths and numbers: the NRD populations bred in isolated valleys present more and shorter ROH segments, while the CSD populations have fewer and longer ROH, which is likely due to the fact that they have undergone more admixture events during the horizontal transhumance practice followed by a more recent standardization. We identified four genes within signatures of selection on chromosome 11 related to fertility in the NRD group, and 23 genes on chromosomes 5 and 6 related to growth in the CSD group. Finally, we identified 17 genes on chromosome 12 related to environmental adaptation and body size with high homozygosity in both groups.

**Conclusions:**

These results show how different management practices have impacted the level of genomic inbreeding in two Italian goat groups and could be useful to assist management in a low-input system while safeguarding the diversity of small populations.

**Supplementary Information:**

The online version contains supplementary material available at 10.1186/s12711-021-00685-4.

## Background

Today, in light of the ongoing climate change, the management and conservation of livestock biodiversity are becoming an increasingly important goal at the global level [1]. To face this challenge, it is crucial to draw a precise picture of the genetic structure of the indigenous breeds and populations of farmed animals in different countries. It is necessary to understand the genetic basis of their adaptation, not only to the natural environment, but also to the breeding conditions and management strategies to which they have been subjected [2]. From this point of view, Italy provides a good model because it is characterized by a rich biodiversity in all domesticated species thanks to its varied history, environment, climate, and farming traditions [3, 4]. Goats, in particular, represent one of the greatest expressions of Italian biodiversity with more than 30 autochthonous breeds and populations reared under very diverse climates and farming conditions, several of which are considered as low-input agricultural systems [5].

In the Northern regions, goats are mainly bred in the Alps, where two diametrically opposed farming systems coexist. On the one hand, in the valleys and hilly regions, modern intensive and semi-intensive farming systems are present, which are specifically suited to milk and cheese production and usually exploit cosmopolitan dairy goat breeds, particularly Saanen and Alpine [6, 7]. In these systems, medium-to-large flocks are mostly kept indoor with controlled feeding and limited grazing, which is generally conducted in fenced pastures near the farm [8]. On the other hand, the traditional extensive farms, which can be considered as low-input/low-output systems, are mainly located in the mountainous areas and depend highly on natural grazing. On these farms, small flocks of local breeds are kept indoor during the winter and in pasture for the rest of the year because of extreme variations in climate and weather conditions, especially during the winter. Some farmers still practice the traditional vertical transhumance (*alpeggio*), which consists in transferring the animals to alpine pastures during the summer only [6–8]. The animals of this farming system are particularly influenced by the climate conditions.

Central-southern Italy and the islands count the largest number of goat farms and heads [9]. In these regions, which are characterized by a hotter and dryer climate [5] than in Northern Italy, the traditional extensive or semi-extensive farms of autochthonous goat breeds prevail and are generally located in marginal mountainous areas [10, 11]. The vertical transhumance was usually combined with a horizontal transhumance; for example, the shepherds transferred all their animals—cattle, sheep, goats, and shepherd dogs—in the mild Apulian plains during the winter and returned to the Abruzzo mountains in the summer [12].

These differences between Northern and Southern Italian goat populations in terms of animal nutrition, housing, and mating management, may have contributed to the genetic makeup of the Italian caprine diversity. Among the genomic tools and approaches that are now proposed to characterize animal biodiversity, the analysis of runs of homozygosity (ROH) is certainly one of the most useful [13]. ROH are long stretches of homozygous genotypes in the genome of an individual, which compose a pair of identical haplotypes. They are considered as a standard approach for the calculation of genomic inbreeding values (*F*_ROH_) and for the detection of signatures of selection [14]. The length of a ROH can also be a useful indicator of the time of the inbreeding event with which it is associated, i.e. long ROH are associated with recent events of inbreeding in the history of a breed or of a single individual, whereas short ROH indicate a more ancient event [15]. The presence of several ROH in a particular region of the genome of a species or a population, regardless of their length, constitutes a so-called ROH island. The analysis of ROH islands can be a very effective tool to identify the regions of a genome that have been under selective pressure because they can contain variants that are shared between the individuals of a specific population [16]. For all these reasons, the analysis of ROH from genomic data and the derived inbreeding value (*F*_ROH_) are increasingly used as a starting point to develop new management systems of animal populations, together with the more traditional pedigree information [17].

In this work, our aim was to characterize ROH in 902 goats from the Northern and Southern Italian groups, estimate their level of inbreeding, and analyze how it has evolved across generations according to management practices.

## Methods

### Dataset and quality control

In this work, we used the same Italian Goat Consortium2 (IGC2) dataset as described in Cortellari et al. [5]. Among the 34 populations present in that dataset, we decided to exclude the Bezoar, which in the previous work was used as an outgroup, the Maltese × Sarda crosses and the two Montecristo populations due to their unique history of isolation (feral) or farming (mainland). All the animals were genotyped with the Illumina Goat single nucleotide polymorphism (SNP)50 BeadChip. SNPs that had a missing genotype frequency higher than 0.2, or that were in unplaced scaffolds or on the X chromosome were excluded from the analysis, but we did not apply a threshold for minor allele frequency (MAF) to better identify ROH [18]. Individuals with a call rate lower than 95% were removed. All quality control procedures were carried out with the software PLINK 1.9 [19]. After the initial quality check on the 986 individuals and the 52,538 SNPs taken from the original IGC2 dataset, 902 animals grouped in 30 breeds and 46,995 SNPs were retained. Population structure of the goats included in the final dataset was investigated by multidimensional scaling analysis (MDS) and by building a phylogeny tree based on Reynolds genetic distances.

### Expected heterozygosity (genetic diversity)

For each breed, the PLINK 1.9 software (-hardy option) was used to calculate the expected heterozygosity (H_E_), observed heterozygosity (H_O_), and Wright’s fixation index (*F*_IS_), which is defined as the correlation between the homologous alleles within an individual relative to the local population to which that individual belongs [20].

### Definition of runs of homozygosity

In order to minimize the discovery of false positives within regions of low marker density, we selected rather stringent criteria [18]. ROH were calculated separately for each individual using the software PLINK 1.9 by applying a sliding window of 20 SNPs. A ROH was called if the following parameters were fulfilled: (i) no heterozygous genotypes, (ii) less than two missing genotypes, (iii) a minimum number of SNPs within a ROH ≥ 20, (iv) a minimum ROH length of more than 1 Mb, (v) a minimum SNP density of two SNPs per Mb, and (vi) a maximum gap of 500 kb between consecutive homozygous SNPs.

### ROH distribution and genomic inbreeding

To characterize the ROH distribution, for each breed we estimated: the number of individuals without ROH, the mean number of ROH per individual, the mean total length of ROH per individual, the mean length of a ROH per individual, and the genomic inbreeding coefficient (*F*_ROH_) for each individual. The *F*_ROH_ for each breed was computed following the method proposed by McQuillan [21]:

$${F}_{ROH, i} = \frac{{L}_{ROH} }{{L}_{AUTO}},$$

where ${L}_{ROH}$ is the sum of the total length of ROH in individual $i$ and ${L}_{AUTO}$ is the total length of the autosomes covered by SNPs. In addition, we categorized the ROH for each breed into five length classes (1–2 Mb, 2–4 Mb, 4–8 Mb, 8–16 Mb, and > 16 Mb) to compare the distribution of the *F*_ROH_ across these categories between the considered breeds [22]. We focused on these length classes with the intent to investigate the percentage and the impact of ancient and more recent inbreeding events that occurred in the Italian goat breeds.

### Identification of the groups of populations

In order to better disentangle the genetic differences between the Italian goat populations analyzed due to climatic conditions and breeding management techniques, we divided them into two large groups according to their geographical distribution: a group of ten populations from the Northern Italy breeds (NRD) and a group of 20 populations from the Central-southern Italy breeds (CSD), which also includes the two Maltese populations (MAL and SAM).

### Statistical analysis

We performed two linear mixed models to evaluate the statistical significance of the difference between the ROH parameters identified for each population group [23] using the statistical software JMP 16 [24]. We modelized two variables ($\mathrm{Y}$): (i) a standardized ratio between the length of single ROH in each individual and the length of the corresponding chromosome $\bigg(std\left(\frac{ROH\,length}{CHR\,length}\right)\bigg)$, and (ii) a standardized ratio between the number of ROH on each chromosome of each individual and the length of the chromosome $\bigg(std\left(\frac{ROH\,number}{CHR\,length}\right)\bigg)$; both of these models included the same factors:

$$\mathrm{Y}=\upmu +\mathrm{CHR}+\mathrm{POPGROUP}*\mathrm{CHR}+\mathrm{BREED}\left[\mathrm{POPGROUP}\right]+\mathrm{POPGROUP}+\mathrm{id}+\mathrm{e},$$

where $\upmu$ is the mean, $\mathrm{CHR}$ is the fixed effect of the autosome (chromosomes 1 to 29), $\mathrm{POPGROUP}$ is the fixed effect of the population group (CSD vs NRD), $\mathrm{BREED}[\mathrm{POPGROUP}]$ is the fixed effect of the breed nested within the population groups (n = 20 in CSD and n = 10 in NRD), $\mathrm{POPGROUP}*\mathrm{CHR}$ is the interaction between population group and autosome, $\mathrm{id}$ is the random effect of the animal, and $\mathrm{e}$ is the random residual. The covariance between the animals was assumed to be equal to 0.

### Signatures of selection

In this work, we investigated the signatures of selection by using three methods: ROH, ΔROH islands and the Wright’s fixation index (*F*_ST_).

For the first method, a homozygosity score (H-score) ranging from 0 (0%) to 1 (100%) was obtained for each SNP by counting how many times it appeared in a ROH and dividing the result by the number of the animals. This approach was applied to each population separately, and the top 1% H-scores were considered. SNPs that were within regions of 0.25 Mb were joined together, and regions with more than 15 SNPs were considered as ROH islands. Then, the identified ROH islands were investigated to list the annotated genes they contain based on the reference genome (ARS1) and the associated functions and pathways.

The ΔROH score was defined as the difference between the H-scores for the CSD and the NRD groups at a specific SNP. The regions of maximum difference in homozygosity were defined analogously to the ROH islands (top 1% values, SNPs within a region of 0.25 Mb combined together, and regions encompassing > 15 SNPs), thus resulting in ΔROH islands.

The Wright’s fixation index (*F*_ST_) was calculated using the PLINK software, averaging the value of each SNP with the values of five adjacent SNPs in each flanking region to minimize the impact of outlier scores [22]. The top 1% averaged *F*_ST_ values were considered and investigated for annotated genes in the reference genome (ARS1) within a region spanning ± 0.25 Mb from each SNP.

Finally, the genes identified by these three methods were analyzed to detect the shared genes.

## Results

### Dataset composition and quality control

The dataset used for the analyses consisted of 902 goats belonging to 30 populations. The number of analyzed animals per breed are in Table 1. The geographic distributions, the MDS plot, and the phylogeny tree of the studied goat populations are reported in Additional file 1: Fig. S1.

**Table 1:** Dataset composition and mean ROH and genetic parameters for the studied Italian goat breeds

Breed ID	Breed name	Raw dataset	Quality checked	ROH number	ROH total length ± se	ROH length ± se	*F*_ROH_	H_E_	H_O_	*F*_IS_
ALP	Camosciata delle Alpi	143	117	31.5	169.1 ± 12.1	5.0 ± 0.2	0.07	0.41	0.40	0.02
ARG	Argentata dell'Etna	48	46	9.1	31.2 ± 5.8	2.9 ± 0.3	0.01	0.41	0.41	0.00
ASP	Capra dell'Aspromonte	24	24	22.6	113.1 ± 29.1	4.2 ± 0.5	0.05	0.40	0.40	0.00
BIA	Bianca Monticellana	24	23	30.8	158.7 ± 34.8	4.0 ± 0.6	0.06	0.40	0.39	0.01
BIO	Bionda dell'Adamello	24	24	22.4	93.5 ± 20.3	3.5 ± 0.4	0.04	0.40	0.40	0.00
CAP	Capestrina	24	22	23.5	117.5 ± 41.2	3.5 ± 0.4	0.05	0.40	0.40	− 0.01
DDS	Derivata di Siria	32	25	37.8	232.2 ± 28.8	5.5 ± 0.5	0.09	0.40	0.38	0.03
FAC	Facciuta della Valnerina	24	24	19.5	167.9 ± 48.3	5.4 ± 0.8	0.07	0.41	0.39	0.03
FUL	Fulva del Lazio	22	20	11.8	63.0 ± 14.2	4.1 ± 0.5	0.03	0.41	0.41	− 0.02
GAR	Garganica	40	37	21.6	118.8 ± 24.1	4.4 ± 0.3	0.05	0.40	0.40	0.00
GCI	Grigia Ciociara	43	39	17.5	116.4 ± 21.4	4.8 ± 0.6	0.05	0.41	0.40	0.02
GIR	Girgentana	59	56	74.5	322.7 ± 24.1	4.1 ± 0.2	0.13	0.36	0.36	0.01
GRF	Garfagnana	28	25	33.1	147.5 ± 23.7	4.1 ± 0.4	0.06	0.40	0.40	0.01
JON	Jonica	16	15	24.3	88.4 ± 16.1	3.4 ± 0.4	0.04	0.37	0.41	− 0.10
LIV	Capra di Livo-Lariana	24	22	20.6	56.8 ± 7.5	2.6 ± 0.2	0.02	0.40	0.40	− 0.02
MAL	Maltese	16	16	70.6	347.5 ± 56.1	4.4 ± 0.4	0.14	0.37	0.36	0.01
MES	Messinese	24	23	9.3	36.8 ± 8.6	3.5 ± 0.6	0.01	0.40	0.41	− 0.01
MON	Capra di Montefalcone	24	23	16.7	136.4 ± 45.2	5.2 ± 0.9	0.06	0.40	0.40	0.01
NIC	Nicastrese	24	24	24.2	170.3 ± 40.3	5.3 ± 0.7	0.07	0.40	0.39	0.02
NVE	Nera di Verzasca	19	19	32.6	136.2 ± 27.2	3.7 ± 0.4	0.06	0.38	0.39	− 0.02
ORO	Orobica	23	23	92.8	293.6 ± 22.9	3.1 ± 0.1	0.12	0.35	0.36	− 0.02
RCC	Roccaverano	28	28	20.7	169.6 ± 37.6	6.1 ± 0.7	0.07	0.41	0.40	0.03
RME	Rossa Mediterranea	46	40	24.2	93.9 ± 12.8	3.4 ± 0.2	0.04	0.39	0.41	− 0.05
SAA	Saanen	44	44	25.8	115.9 ± 10.1	4.2 ± 0.2	0.05	0.41	0.41	− 0.01
SAM	Maltese sampled in Sardinia	15	15	57.1	294.4 ± 49.0	4.8 ± 0.5	0.12	0.36	0.37	− 0.03
SAR	Sarda	33	33	18.1	77.8 ± 16.7	3.4 ± 0.4	0.03	0.41	0.40	0.01
TER	Capra di Teramo	43	30	25.2	234.1 ± 36.1	7.3 ± 0.7	0.10	0.39	0.38	0.01
VAL	Valdostana	24	24	50.9	272.0 ± 49.5	4.7 ± 0.5	0.11	0.37	0.37	0.02
VLS	Vallesana	24	17	76.6	364.6 ± 45.6	4.7 ± 0.4	0.15	0.36	0.35	0.02
VPS	Capra della Val Passiria	24	24	23.1	118.3 ± 24.3	4.4 ± 0.5	0.05	0.40	0.40	0.01

*F*_ROH_: ROH-based inbreeding; ROH total length ± se in Mb; ROH length ± se in Mb; H_E_: expected heterozygosity; H_O_: observed heterozygosity; *F*_IS_: Wright’s fixation index

### ROH description and genetic diversity

In total, 28,383 ROH were identified in the 902 individuals considered; five animals displayed no ROH: one Garganica individual, one Rossa Mediterranea individual, one Grigia Ciociara individual, and two Capra di Teramo individuals. In terms of average number of ROH per animal, the breed with the largest number was Orobica (92.8 ROH per individual), followed by Vallesana (76.6), both of these breeds being raised in the northern regions of Italy. The breeds with the smallest average number of ROH were two Sicilian breeds, Messinese and Argentata dell’Etna with 9.3 and 9.1 ROH per individual, respectively. Argentata dell’Etna and Messinese were also the breeds that, together with the Val di Livo (or Lariana) had the lowest average value of the total ROH length per individual (31.2, 36.8, and 56.8 Mb, respectively), while the two highest values were found in Vallesana (364.6 Mb) and Maltese bred in Sicily (347.5 Mb). When the average length of ROH per individual in each breed was considered, the Capra di Livo-Lariana, Argentata dell'Etna, and Orobica were the breeds with the lowest values, whereas the Capra di Teramo and Roccaverano were those with the highest values (Table 1).

**Fig. 1:**
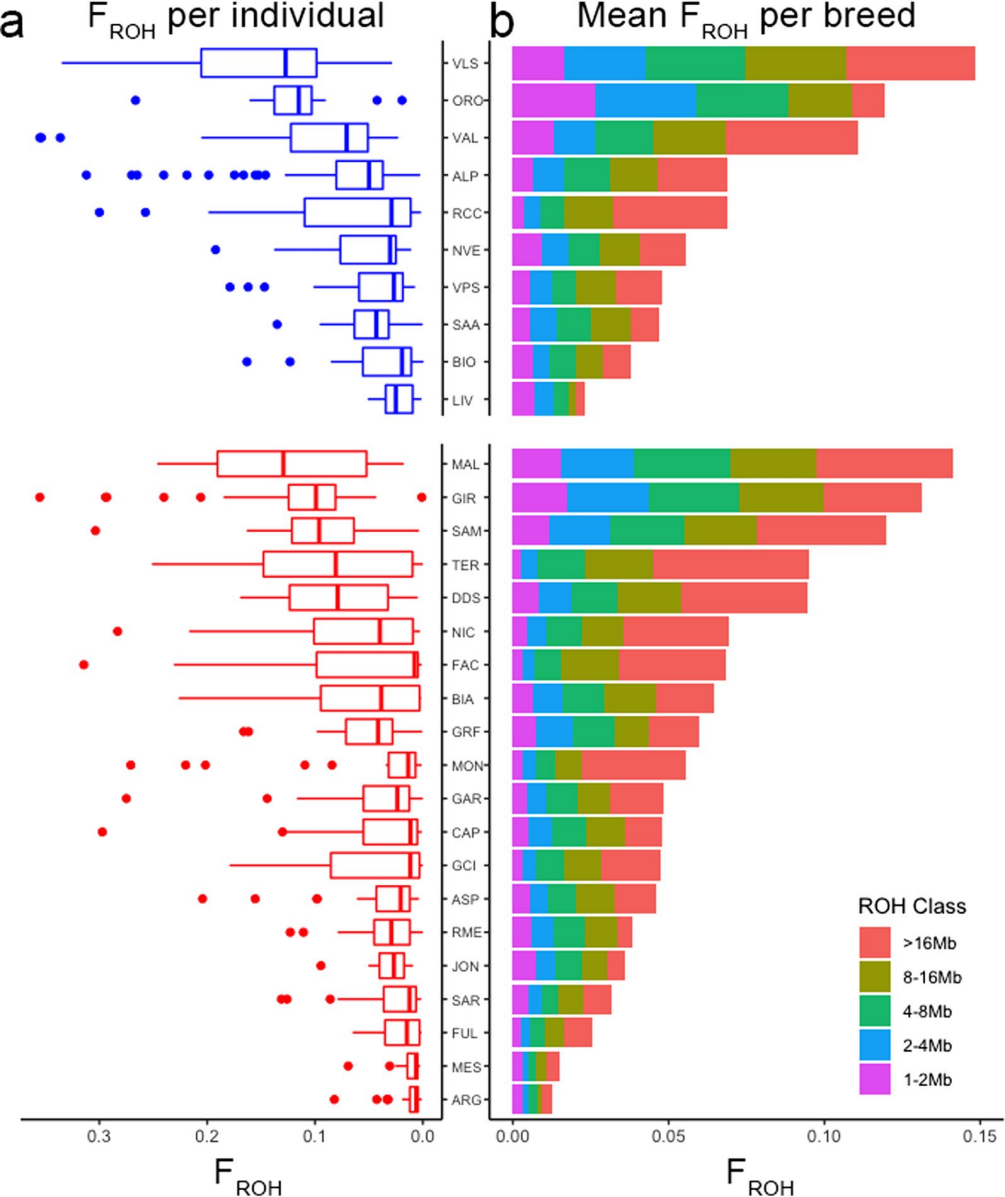
Distribution of *F*_ROH_ values per individual and mean values per breed. Boxplot (**a**) of the single individual *F*_ROH_ distribution in each population (Northern breeds: blue boxplots, Central-southern breeds: red boxplots) with matching barplot (**b**) of the mean *F*_ROH_ values (each color representative of the different ROH length classes)

The ROH-based inbreeding values (*F*_ROH_) showed that the two breeds with the highest level of inbreeding were Maltese and Vallesana and those with the lowest levels were Messinese, Argentata dell’Etna, and Capra di Livo-Lariana. However, the distribution of the individual *F*_ROH_ within each population varied among breeds (see the boxplot in Fig. 1): some breeds such as Maltese, Capra di Teramo, Vallesana, and Girgentana showed a wide dispersion of the individual inbreeding values, while other breeds such as Saanen, Rossa Mediterranea, Capra di Livo-Lariana, and Messinese showed a more compact distribution. The genomic diversity parameters were similar across all the breeds, with the lowest H_E_ and H_O_ respectively in the Orobica (0.35) and Vallesana (0.35), and the highest ones in Roccaverano (0.41) and Saanen (0.41). The *F*_IS_ highest values were found for Roccaverano and Derivata di Siria, and the lowest for the Jonica and Rossa Mediterranea breeds (Table 1).

The ROH identified in all the populations were classified into the five length classes. The largest numbers of identified ROH belonged to the 1–2 Mb (11,294) and the 2–4 Mb (7525) classes. Nevertheless, the length classes that contributed most to the calculated inbreeding value (*F*_ROH_) in the different populations were 8–16 Mb and > 16 Mb. Analysis of the distribution of the ROH categories among all the populations and of their proportional weight in the definition of the mean *F*_ROH_ value revealed three categories: (1) one for which the influence of the longest ROH on the total *F*_ROH_ value appeared to predominate, such as for the Capra di Teramo, Roccaverano, and Montefalcone breeds; (2) one for which the different ROH length classes were well balanced, such as for the Girgentana, Bianca Monticellana, and Nera di Verzasca breeds; and (3) one for which the short ROH were more important, such as for the Orobica, Capra di Livo-Lariana, and Rossa Mediterranea breeds (Fig. 1).

### Statistical analysis

**Fig. 2:**
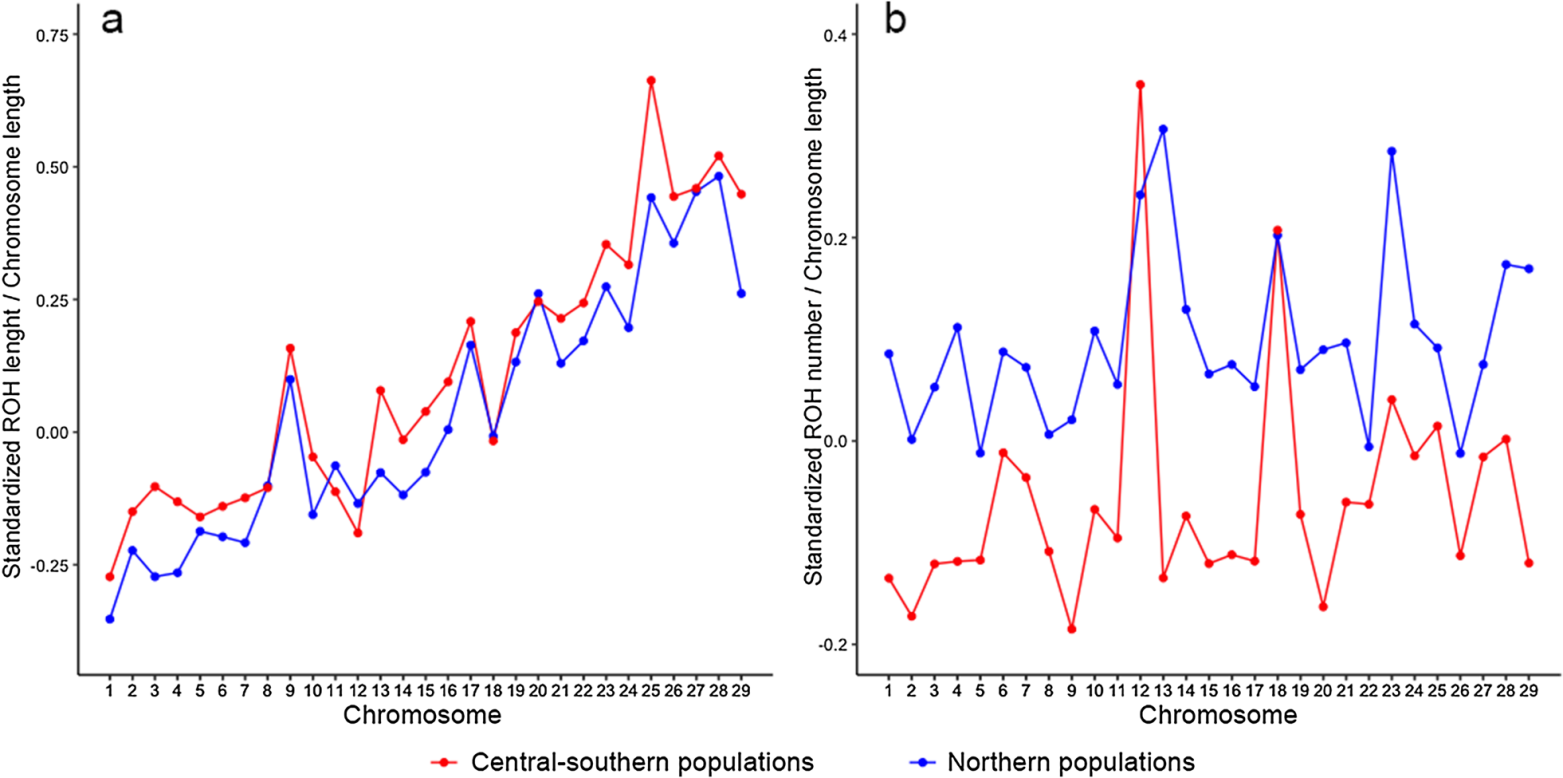
Comparison of mean standardized length and number of ROH in the two groups of Italian goat populations. Graphic representation of the mean standardized length (**a**) and number (**b**) of ROH divided by the corresponding chromosome length in the two groups of Italian goat populations

The two groups of Italian goat populations included 560 individuals belonging to 20 breeds for the Central-southern (CSD) and 342 individuals belonging to ten breeds for the Northern (NRD) groups. The statistical models showed a significant difference in the number and length of ROH between the two groups. Particularly, the first model (r^2^ = 0.18) showed that ROH length was significantly affected by the group (p = 0.003), the breeds within each group (p < 0.0001), and the chromosome (p < 0.0001). ROH were longer for the CSD than the NRD group (LSmean ± SE = 0.06 ± 0.02 vs − 0.02 ± 0.02). Figure 2a shows the mean standardized ratio between the length of ROH and the length of the corresponding chromosome $std\left(\frac{ROH\,length}{CHR\,length}\right)$ for each chromosome in the two groups (CSD and NRD): the largest differences were found for chromosomes 3, 13, 25, and 29 and the smallest differences for chromosomes 8, 18, and 20. Interestingly, the smaller chromosomes presented relatively longer ROH in both groups. Previous studies on plants, yeasts, and humans have shown that recombination rates are inversely correlated with chromosome length, which could be due to the lower frequency of multiple crossovers within a chromosome [25–27]; this has also been reported in goats [28] and other species [29].

When the individual number of ROH per chromosome was modelled (r^2^ = 0.40), all the selected factors (group, breed within each group, chromosome, and the interaction between chromosome and group) were significant (p < 0.0001). ROH number was, on average (± SE), larger for the NRD group (0.19 ± 0.03) than the CSD group (− 0.07 ± 0.02). Figure 2b shows the mean standardized ratio between the number of ROH per chromosome and the corresponding chromosome length $std\left(\frac{ROH\,number}{CHR\,length}\right)$ in the two groups (CSD and NRD): the largest differences were found for chromosome 13 and the smallest for chromosomes 8, 18, 20, and 27. It is worth mentioning that the number of ROH for chromosome 12 was large in both groups, particularly in the CSD group.

### Signatures of selection

For both the CSD and NRD groups, we identified the genomic regions with the highest level of homozygosity, corresponding to the top 1% of SNPs (H-score value > 0.107 for the CSD and > 0.116 for the NRD group) and found ten regions distributed on seven chromosomes for the CSD group and 15 regions distributed on nine chromosomes for the NRD group. Among these regions, six were partially or totally shared because they were highly homozygous in both groups, while the remaining 13 were specific to only one of the two groups. Matching the positions of these regions with those of the genes annotated in the goat genome version ARS1 and excluding genes for which a symbol or orthologs were not available (i.e. beginning with “LOC”) and transfer RNA gene sequences (TRNA), we identified 133 genes specific to the NRD group, 47 genes specific to the CSD group, and 111 genes common to the two groups.

Then, we identified the regions that showed the largest difference in homozygosity between the two groups (ΔH-score values > 0.06) and found nine regions that were distributed on seven chromosomes and harbored 80 genes of the 291 previously identified genes. These regions were both highly homozygous within a group and capable of differentiating the two NRD and CSD groups.

**Fig. 3:**
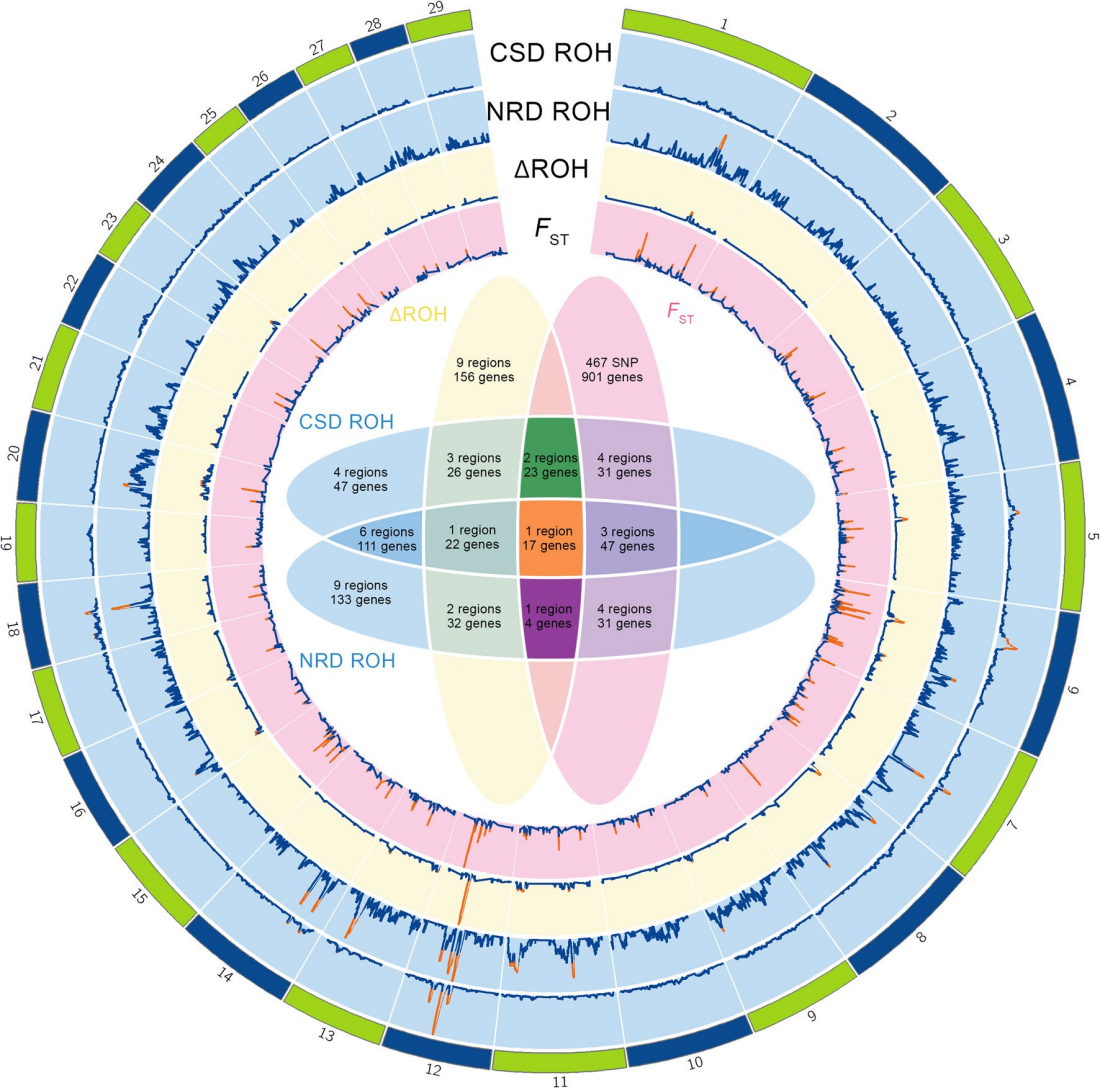
Circos plot of the analysis of signatures of selection with a Venn diagram of the results. Circos plot of the signatures of selection in the two groups of Italian goat populations (external blue tracks), of the ΔROH (middle yellow track) and averaged *F*_ST_ (inner red track). The Venn diagram shows the number of regions and genes shared across methods. *CSD* Central-southern population group, *NRD* Northern population group

Finally, when the results of the previous analyses were cross-referenced with the top 1% mean *F*_ST_ values (> 0.09), we identified 44 genes that were shared among all the genes detected by the three methods (Fig. 3). In particular, two groups of genes were specific to the CSD group, i.e. one on chromosome 6 and one on chromosome 5, and one group specific to the NRD group on chromosome 11; finally, a gene cluster on chromosome 12 that was revealed by the *F*_ST_ analysis was highly homozygous in both groups and had a high ΔROH score (Fig. 3 and see Additional file 2: Fig. S2). The complete list of the identified genes is in Additional file 3: Table S1.

## Discussion

Italy is characterized by a wide variety of breeding environments, managements, and traditions; the effect of this variability is particularly evident in goats, which have been traditionally bred in low-input systems, which are strongly affected by climatic conditions. In our study, we used the well-established genomic tool of runs of homozygosity to shed light on the impact of different management practices on Italian goat homozygosity.

Our results show that some populations present an extremely small number of ROH per individual and, consequently, a level of genomic inbreeding near zero. Among these, the two Sicilian breeds Argentata and Messinese are known to have been crossbred, mainly due to the typical management of traditional extensive farms that share common pastures [30]. Another interesting breed is the Capra di Livo-Lariana, with a very low level of inbreeding that can be explained by historical events, including introgression of many unknown individuals from the surrounding valleys.

Another fundamental aspect that emerges from our work is the possibility of monitoring the inbreeding management in the populations through the evaluation of the relationship among the various ROH length classes. Indeed, regardless of the absolute value of *F*_ROH_, the populations that have a large preponderance of long ROH (> 16 Mb) are more likely to have been under a non-optimal management with frequent mating between closely related individuals, a possible consequence of the reduced number of individuals in the population. One example is the Capra di Teramo breed: the earthquakes that hit the regions where this breed is reared had catastrophic consequences on this already endangered population. On the contrary, Orobica has a more balanced ratio between the different ROH length categories and a smaller number of long ROH. In fact, Orobica is one of the first Italian breeds to have been standardized and reflects a long-term efficient management and the particular attention paid by shepherds to the mating plans.

Moreover, the statistical models performed on ROH number, length, and distribution revealed significant differences between the two NRD and CSD groups. In particular, for the NRD group the number of ROH per individual was larger than for the CSD group, whereas for the CSD group the ROH were longer than for the NRD group. The populations from the NRD group have always been bred in isolated valleys, with natural barriers that prevent the exchange of animals; for this reason, a large number of short ROH, indicating ancient inbreeding events, is expected. On the contrary, the breeds from the CSD group might have undergone, in the past, more admixture events due to the horizontal transhumance practice, the sharing of common pastures, and the presence of multi-breed farms; the more recent standardization is represented by longer ROH.

We also identified signatures of selection that characterize Italian goat populations according to their geographic location. For the NRD group, only four genes on chromosome 11 were found across all the analyses. Among these, the most interesting one is *DENND1A*, a fertility-related gene [31] that is involved in embryogenesis in cattle. Furthermore, other genes worthy of attention belong to highly homozygous regions but were not found by the ΔROH analysis. In particular, two genes on chromosome 11, *HSPA5* and *NR5A1,* are linked to the production of the anti-Müllerian hormone in grazing cows [32] and are located in a large region on this chromosome that is related to milk production in European, American, and Asian goats [33]. Another region of interest is located on chromosome 13 and hosts genes that are important for pigmentation such as *ASIP* and *RALY* [34].

We found a particularly interesting group of genes for the CSD group in a region of chromosome 6 that distinguishes it from the NRD group. This region harbors different genes related to animal growth and development, such as *LCORL* [35], which has been shown to regulate body size in goats and several other mammals, *HERC6* [36], and *FAM184B* [37]. A part of this homozygous region and another region that we also found on chromosome 5 for the CSD group were previously described by [30], who analyzed only the cluster of Sicilian goats.

Finally, in both NRD and CSD groups, we identified a highly homozygous region on chromosome 12 that was detected by all three analyses and contains 17 genes, which are mostly related to environmental adaptation, for example to hot and arid climates, and body size, including *GJB2*, *GJA3* [38], and *PSPC1* [39].

## Conclusions

Our findings show that the analysis of ROH is a useful tool not only to identify regions under selection in different breeds, but also to evaluate how their management has evolved over generations. However, this is possible only if a representative recent sample of the specific populations is available, with the potential of expanding the study to historical samples to understand the evolution of a breed’s inbreeding and signatures of selection. ROH assessment can be adopted as a ‘checkpoint’ to assess whether selection in a population is leading to an increase in its average homozygosity and inbreeding, therefore indicating whether a fine-tuning of the breeding scheme is necessary. For these reasons, we recommend the implementation of this tool in the routine evaluation of biodiversity and, consequently, the management of autochthonous populations that are bred in a low-input system as typical of marginal rural areas.

## Supplementary Information

**Additional file 1: Figure S1.** Geographic distribution (**a**), phylogeny tree (**b**), and multidimensional scaling analysis (**c**) of all the Italian goat breeds included in the study.

**Additional file 2: Figure S2.** Manhattan plots representing the signals of signatures of selection in the two population groups (CSD and NRD), of the ΔROH, and averaged *F*_ST_. CSD = Central-southern population group; NRD = Northern population group.

**Additional file 3: Table S1.** List of the genes identified by the analyses of the signatures of selection: top 1% homozygosity score in CSD and NRD groups, ΔROH, and averaged *F*_ST_. CSD = Central-southern population group; NRD = Northern population group.

## Data Availability

The datasets generated and/or analyzed during the current study are available on Mendeley Data (https://doi.org/10.17632/hnd59x6gmg.1; URL: https://data.mendeley.com/datasets/hnd59x6gmg/1).
