# Supplementary material for: Runs of homozygosity in the Italian goat breeds: impact of management practices in low-input systems
Source: Genet Sel Evol. 2021 Dec 11;53:92. doi: 10.1186/s12711-021-00685-4 (PMC8666052; doi:10.1186/s12711-021-00685-4)

Figure S1. Geographic distribution (a), phylogeny tree (b), and multidimensional scaling analysis (c) of all the Italian goat breeds included in the study.

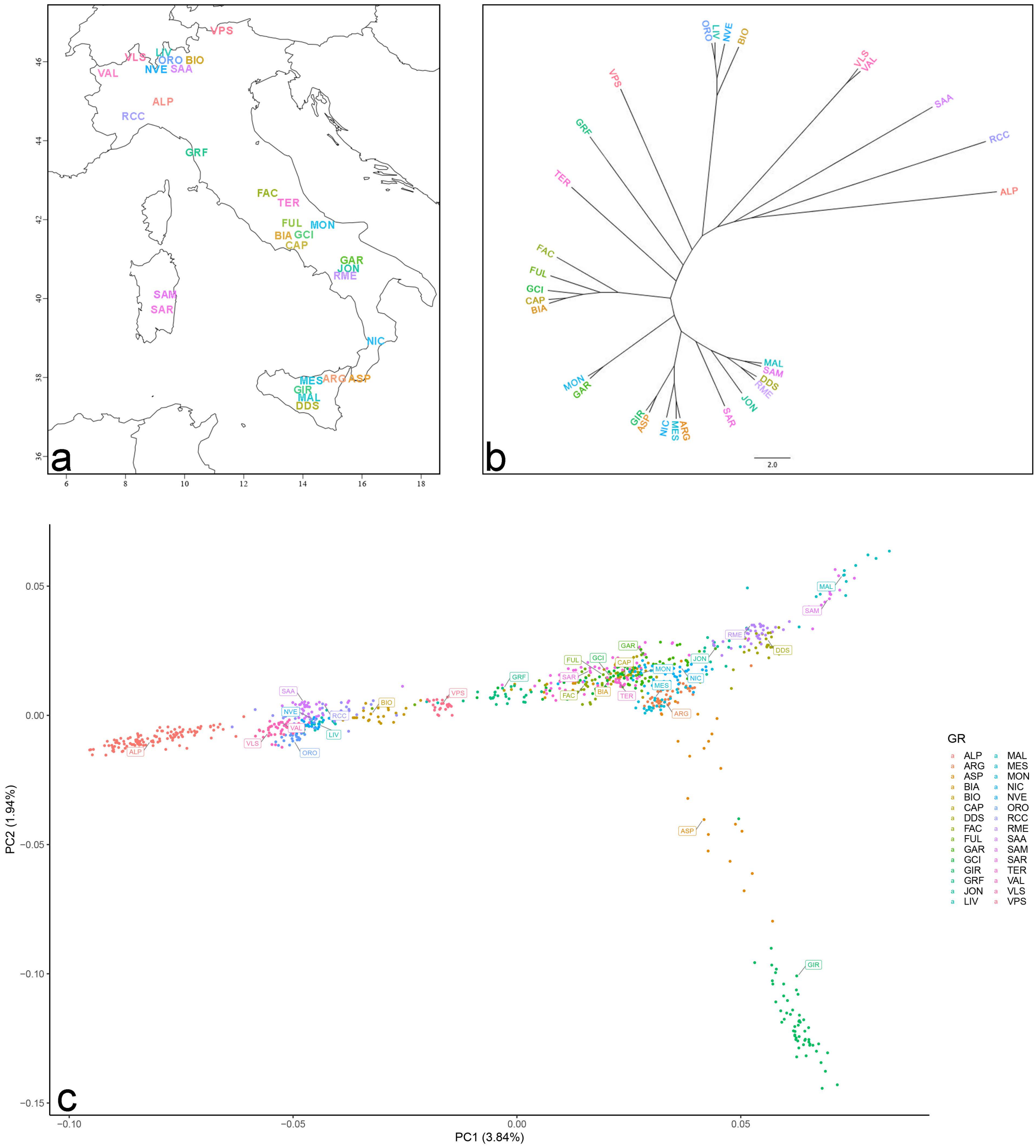

Supplement: Supplementary file 1 — Additional file 1: Figure S1. Geographic distribution (a), phylogeny tree (b), and multidimensional scaling analysis (c) of all the Italian goat breeds included in the study. [file 12711_2021_685_MOESM1_ESM.pdf]
