# Supplementary material for: Runs of homozygosity in the Italian goat breeds: impact of management practices in low-input systems
Source: Genet Sel Evol. 2021 Dec 11;53:92. doi: 10.1186/s12711-021-00685-4 (PMC8666052; doi:10.1186/s12711-021-00685-4)

Figure S2. Manhattan plots representing the signals of signatures of selection in the two population groups (CSD and NRD), of the ΔROH, and averaged F_{ST}. CSD = Central-southern population group; NRD = Northern population group.

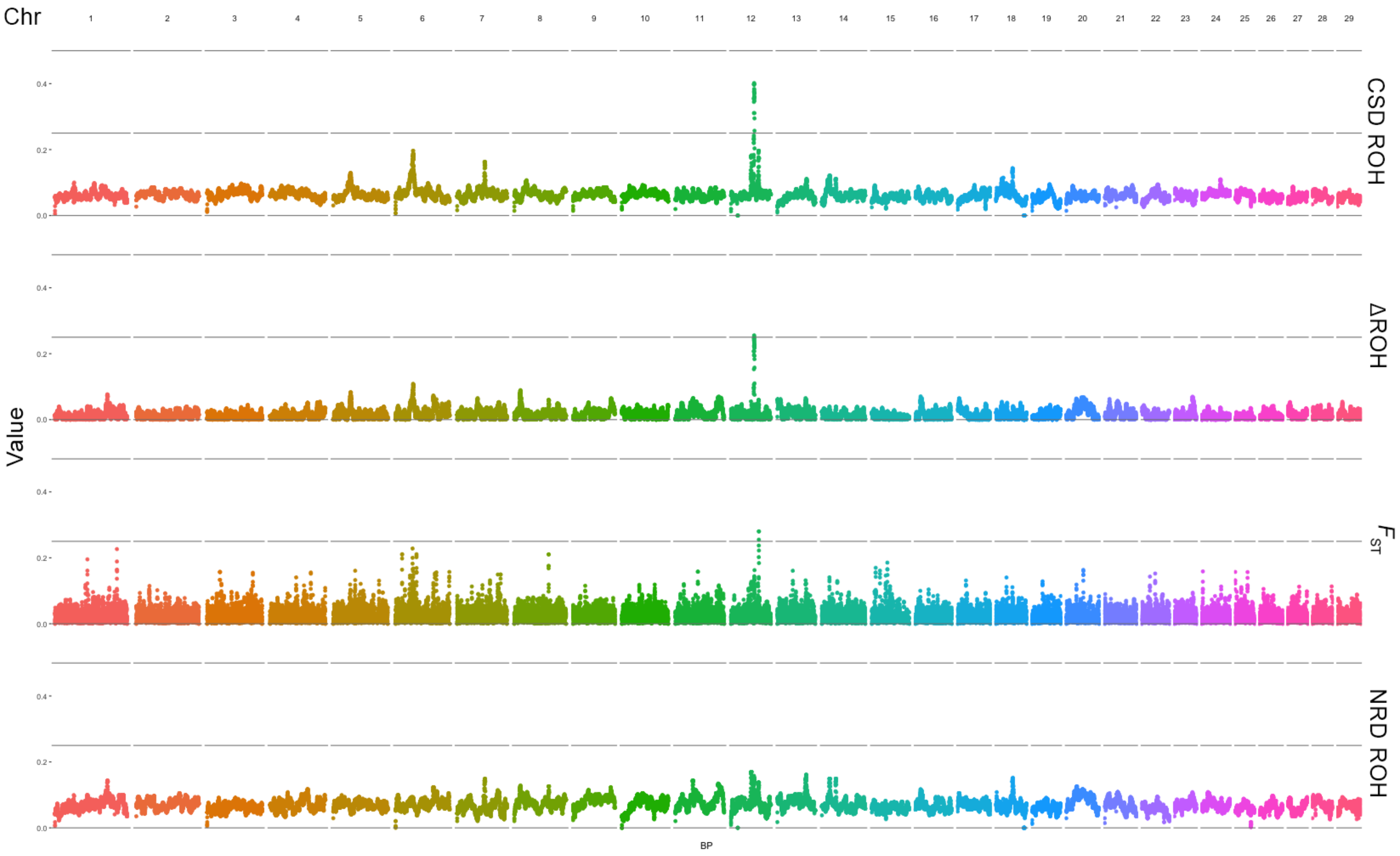

Supplement: Supplementary file 2 — Additional file 2: Figure S2. Manhattan plots representing the signals of signatures of selection in the two population groups (CSD and NRD), of the ΔROH, and averaged FST. CSD = Central-southern population group; NRD = Northern population group. [file 12711_2021_685_MOESM2_ESM.pdf]
